# Global Transcriptomic Analysis Identifies Gene Expression Programs Regulated by α-Factor in Yeast

**DOI:** 10.4014/jmb.2509.09015

**Published:** 2025-12-18

**Authors:** Soojin Yeom, Jung-Shin Lee

**Affiliations:** 1 Department of Molecular Bioscience, College of Biomedical Science, Kangwon National University, Chuncheon 24341, Republic of Korea; 2 Institute of Life Sciences, Kangwon National University, Chuncheon 24341, Republic of Korea; 3 Department of Biomedical Sciences, Yonsei University College of Medicine, Seoul, Republic of Korea; 4 Human-Inspired AI Research, Korea University, Seoul, Republic of Korea

**Keywords:** *Saccharomyces cerevisiae*, α-factor, pheromone signaling, cell cycle arrest, chromatin remodeling

## Abstract

In yeast, the mating pheromone α-factor triggers a signaling cascade in haploid "a" cells, leading to G1 cell cycle arrest, polarized growth ("shmoo" formation), cell wall remodeling, and ultimately cell fusion. While the physiological responses to α-factor are well established, a genome-wide analysis of transcriptional changes in response to α-factor has not been previously reported. Here, we performed RNA sequencing (RNA-seq) to profile gene expression changes in "a" mating type yeast cells treated with α-factor. We identified 957 differentially expressed genes (DEGs), including 448 upregulated and 509 downregulated genes. Gene ontology (GO) analysis revealed enrichment of upregulated genes in pheromone signaling, cell wall biogenesis, and cell shape regulation. In contrast, downregulated genes were mainly associated with cell cycle progression, chromatin remodeling, histone gene expression, and nucleosome organization. Our dataset validates known pheromone-responsive genes and identifies novel candidates potentially involved in mating responses. These findings provide a valuable resource for understanding how transcriptional regulation and chromatin dynamics are coordinated during pheromone signaling in yeast.

## Introduction

The budding yeast *Saccharomyces cerevisiae* has been widely used as a powerful eukaryotic model organism for elucidating the complex mechanisms of signaling pathways [1]. Among these, the mating pheromone response pathway is one of the best-characterized signaling pathways in yeast. Haploid yeast cells exist in two distinct mating types, "a" (MATa) and "α" (MATα). MATα cells secrete the α-factor, a peptide pheromone that activates a signaling cascade of the opposite mating type, MATa cells [2]. In response to α-factor, MATa cells undergo coordinated molecular and physiological changes essential for mating, such as G1 cell cycle arrest and morphological remodeling [3-7].

Mating in yeast occurs through the fusion of haploid cells of opposite mating types, forming diploid cells [8-13]. This mating process is initiated by mating pheromones: MATa cells secrete a-factor, and MATα cells secrete α-factor. These pheromones selectively recognize cells of the opposite mating type and activate signaling pathways that trigger the physiological changes required for successful mating [2].

Upon recognizing α-factor, MATa cells activate the receptor Ste2, a plasma membrane-localized G protein-coupled receptor (GPCR) [13-16]. Ligand binding to Ste2 initiates an intracellular signaling cascade via the heterotrimeric G protein complex, composed of Gpa1 (Gα), Ste4 (Gβ), and Ste18 (Gγ) subunits. [2, 5, 13-16]. This activation subsequently promotes the recruitment of the scaffold protein Ste5, which assembles the mitogen-activated protein kinase (MAPK) cascade consisting of Ste11 (MAPKKK), Ste7 (MAPKK), and Fus3/Kss1 (MAPK) [15-17]. This MAPK module ultimately leads to the phosphorylation and activation of transcription factors such as Ste12 [13, 16, 18-20]. Activated Ste12 binds to pheromone response elements (PREs) within target gene promoters and, together with transcriptional co-activators such as Mcm1, regulates the expression of genes involved in mating and cell cycle control [3, 16, 21]. Consequently, α-factor stimulation orchestrates extensive transcriptional reprogramming to drive mating-specific responses.

Physiologically, α-factor induces G1 phase cell cycle arrest through the stabilization of the cyclin-dependent kinase inhibitor Far1, which inhibits G1 cyclins (Cln1, Cln2, and Cln3) and prevents progression into S phase [19, 22-25]. In parallel, cells undergo cytoskeletal reorganization and polarized growth toward the pheromone source, forming the characteristic "shmoo" morphology [16, 26, 27]. Additionally, the cell wall and plasma membrane undergo extensive remodeling through the action of enzymes and structural proteins involved in cell wall biogenesis and modification [13, 16, 28-31].

Importantly, α-factor-induced G1 arrest also influences chromatin organization by preventing entry into S-phase. Histone protein synthesis is tightly coupled to DNA replication during S-phase [32-35], G1-arrested cells fail to produce new histones, leading to insufficient nucleosome assembly and impaired chromatin remodeling. This disruption in histone supply ultimately affects nucleosome dynamics and overall chromatin integrity.

Although the cell cycle has been extensively studied in *S. cerevisiae*, genome-wide transcriptomic analysis specifically addressing the transcriptional impact of pheromone-induced G1 arrest remains limited. In particular, how mating pheromones such as α-factor globally modulate gene expression during this process is not well understood. Despite the frequent use of wild-type (WT) strains in molecular and cellular genetics, a systematic transcriptomic analysis of α-factor responses in these strains has yet to be conducted.

Given that α-factor triggers global transcriptional reprogramming alongside profound physiological remodeling, transcriptome-wide profiling of WT cells during G1 arrest is crucial for understanding how environmental cues interface with chromatin regulation and cell cycle control. `

In this study, we performed what we believe to be the most comprehensive transcriptome-wide analysis to date of a WT strain in response to α-factor treatment using RNA sequencing (RNA-seq). While previous studies have examined pheromone-induced transcription using microarrays [36] or RNA-seq under specialized conditions such as cell-cycle synchronization [37], these earlier datasets were generated under experimental contexts that differ substantially from physiological α-factor treatment. Our analysis quantitatively defined global gene expression changes upon pheromone stimulation, validating known pheromone-responsive genes and identifying novel candidate genes and pathways involved in pheromone signaling, cell cycle arrest, nucleosome assembly, chromatin remodeling, and cell wall remodeling.

This dataset provides an essential reference for understanding α-factor-mediated transcriptional dynamics in WT cells and serves as a valuable resource for future studies on yeast mating responses, cell cycle regulation, and chromatin biology. These findings are expected to contribute broadly to our understanding of pheromone signaling and its impact on cellular physiology, not only in yeast but also in other eukaryotic organisms.

## Methods

### Yeast Strains Used in This Study


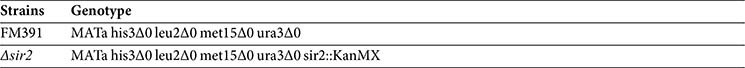


### Yeast Cell Culture and α-Factor Treatment

Yeast cells were grown overnight in YPD medium at 30°C. The overnight culture was diluted to an OD_600_ of 0.1 in fresh YPD and incubated at 30°C with shaking until reaching an OD_600_ of 0.2. α-Factor (final concentration: 9.6 μg/ml, prepared by adding 30 μl of a 16 mg/ml stock solution to 50 ml of culture) was then added to the medium, and cells were incubated for 1.5 h at 30°C with continuous shaking. A second α-factor treatment was performed under the same conditions to ensure sustained pheromone exposure. Following treatment, cells were harvested by centrifugation and washed twice with sterile distilled water prior to further analysis.

### RNA Preparation

Following α-factor treatment, yeast cells were harvested by centrifugation when the culture reached an OD_600_ of approximately 1.0. Cells were subjected to enzymatic digestion with zymolyase to disrupt the cell wall. Total RNA was then extracted using the NucleoSpin RNA Kit (Macherey-Nagel, Cat. No: 740955, Germany) according to the manufacturer’s in 258010 structions.

### RNA Sequencing (RNA-seq) and Data Analysis

RNA-seq progressed with biological duplication. The library for sequencing was prepared following the manufacturer's instructions of NEBNext Ultra II Directional RNA Library Prep Kit for Illumina (NEB, Cat. No: E7760, USA). Sequencing was performed on an Illumina Hi-seq 2500 instrument to generate 101 bp paired-end reads for each sample. Trimmomatic v.0.36 performed the sequencing adapter removal and quality-based trimming on raw data with TruSeq adapter sequences [51]. Cleaned reads were mapped to the *S. cerevisiae* reference genome (SacCer3) using TopHat2 v.2.0.11 with the default parameter [51, 52]. Feature count was used for counting to measure the expression level of each gene, and data were normalized by DESeq2 [53, 54]. For visualization, z-scores were calculated for each gene by subtracting the mean and then dividing by the standard deviation. The pheatmap R package (https://cran.r-project.org/web/packages/pheatmap) was used to perform hierarchical clustering analysis and draw heatmap. We used The Database for Annotation, Visualization and Integrated Discovery [55] to analyze the gene ontology of the differentially expressed genes [56, 57]. We used Integrative genomics viewer (IGV) to visualize the mapped reads upon each gene in the wild-type strains and *sir2Δ* mutant [58]. All genomic data are publicly available at the NCBI GEO repository with the accession number [GSE303575].

### Flow Cytometry Analysis of DNA Content

Cells were harvested and resuspended in 250 μl RNase A solution (0.25 μg/μl in 50 mM Tris-HCl buffer, pH 7.5) and incubated at 55°C for 2 h to degrade residual RNA. Following incubation, cells were washed twice with 100 μl of the same Tris-HCl buffer. The cell pellet was then resuspended in 100 μl pepsin solution (5 μg/μl in 4.5 mM HCl) and incubated at 37°C for 1 h. After pepsin treatment, cells were washed twice with 50 mM Tris-HCl buffer (pH 7.5), resuspended in 100 μl of the same buffer, and briefly sonicated to disperse aggregates. For DNA staining, cell suspensions were transferred to flow cytometry tubes containing 400 μl of SYTOX Green (Molecular Probes; final concentration: 5 μM). DNA content was analyzed using a FACSymphony flow cytometer (BD Biosciences, USA).

## Results

### Expression of 957 Genes Is Altered in Response to α-Factor in “a” Mating Type Cells

**Fig. 1:**
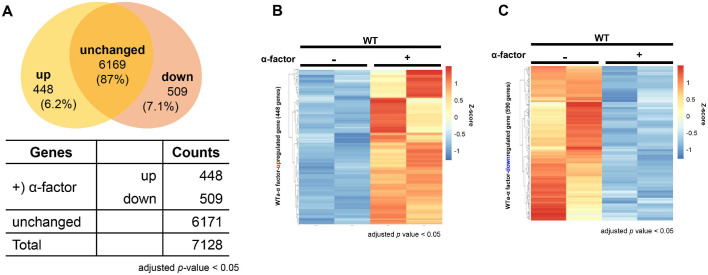
Expression of 957 genes is changed in response to α-factor in WT. (**A**) Venn diagram of differentially expressed genes in WT in response to α-factor treatment. RNA-seq analysis identified 7,128 expressed genes in WT cells. Of these, 957 genes were significantly differentially expressed following α-factor treatment (adjusted *P* < 0.05; log_2_FC > 1 or < –1), including both 448 upregulated and downregulated genes. The Venn diagram illustrates the classification of total expressed genes into three groups: significantly upregulated, significantly downregulated, and non-differentially expressed genes, based on gene expression before and after α- factor stimulation. (**B-C**) Heatmaps of upregulated and downregulated genes in WT after α-factor treatment. Heatmap showing the differential expression patterns of 448 genes significantly upregulated (adjusted *P* < 0.05; log_2_FC > 1) and 509 downregulated in WT cells following α-factor treatment (adjusted *P* < 0.05; log_2_FC <-1). Gene expression values were normalized and visualized using Z-scores. The color scale represents relative expression levels, with red indicating higher expression and blue indicating lower expression. Hierarchical clustering was performed to group genes with similar expression patterns in response to α-factor stimulation.

Budding yeast cells exposed to the mating pheromone α-factor undergo G1-phase cell cycle arrest, a well-established physiological response essential for successful mating [23, 38]. To systematically investigate how α-factor influences gene expression in WT cells, we performed transcriptome-wide analysis using RNA-seq on cells before and after α-factor treatment (Fig. 1). This approach allowed us to comprehensively assess the transcriptional changes associated with pheromone exposure under well-controlled experimental conditions.

Differential expression analysis revealed that α-factor stimulation leads to substantial transcriptional reprogramming in WT cells, characterized by both upregulation and downregulation of distinct gene sets (Fig. S1A). Among the total 7,128 genes analyzed, 448 genes were significantly upregulated, exhibiting at least a two-fold increase in expression upon α-factor treatment. In contrast, 509 genes showed a significant downregulation, with expression levels decreasing by at least two-fold. The expression of the remaining 6,171 genes did not display any notable changes under the same conditions (Fig. 1A).

To further illustrate the global impact of α-factor treatment on gene expression, we performed hierarchical clustering and generated heatmaps of differentially expressed genes (DEGs). These analyses clearly visualized the distinct transcriptional profiles between untreated and α-factor-treated cells, confirming that α-factor stimulation induces a marked genome-wide shift in gene expression (Fig. 1B and 1C). The observed changes were highly consistent with the established roles of the mating pheromone signaling pathway, particularly in regulating cell cycle arrest and driving physiological adaptations necessary for mating.

Importantly, our results confirmed the expected expression patterns of previously characterized pheromone-responsive genes, demonstrating the reliability of our experimental design and analysis. In addition, this genome-wide dataset identified novel genes that had not been previously associated with pheromone signaling, suggesting that additional, yet unexplored, regulatory mechanisms may contribute to the cellular response to α-factor.

Together, these findings provide a detailed and comprehensive view of the transcriptional landscape of WT yeast cells during pheromone-induced G1 arrest. The dataset generated in this study serves as an important resource for future investigations into the molecular mechanisms that govern pheromone signaling, cell cycle regulation, and mating-associated cellular responses in budding yeast.

### α-Factor Induces Transcriptional Activation of Genes Involved in Pheromone Response, Cell Wall Remodeling, and Morphogenesis in Yeast

When budding yeast cells are exposed to mating pheromones such as α-factor, the expression of pheromone response genes is significantly upregulated, leading to characteristic morphological changes, including shmoo formation [4-7, 19]. To systematically investigate these transcriptional alterations, we performed RNA-seq to profile the expression of genes involved in pheromone response and cell wall remodeling. Our RNA-seq analysis identified 448 genes that were significantly upregulated in response to α-factor treatment.

**Fig. 2:**
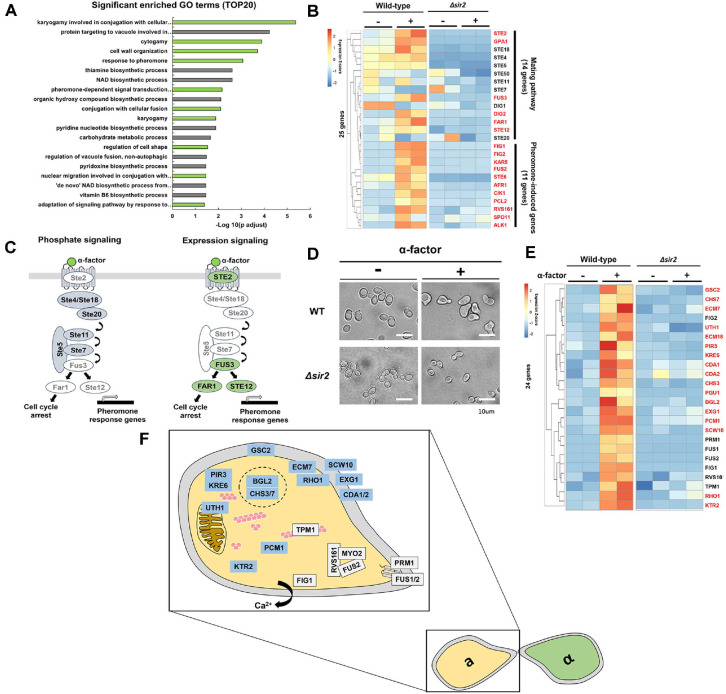
Differential gene expression and Gene Ontology of upregulated genes in WT after α-factor addition. (**A**) Gene ontology (GO) enrichment analysis of upregulated genes in WT after α-factor treatment. GO enrichment analysis was conducted using DAVID on 448 genes that were significantly upregulated in WT cells following α-factor treatment (adjusted *P* < 0.05; log_2_FC > 1). The bar plot shows the top 20 significantly enriched GO categories (biological process) based on statistical significance. The y-axis represents GO terms, and the x-axis indicates –log_10_(**P**) values. Enriched categories include pheromone-dependent signaling, cell wall organization, and mating-related morphogenesis. (**B**) Heatmap of pheromone-responsive genes associated with MAPK signaling in WT. A heatmap displaying the expression patterns of 25 selected genes involved in the pheromone response and mating-related MAPK signaling pathway. These genes were significantly upregulated in WT after α-factor treatment. Gene expression levels are shown as Z-scores of normalized RNA-seq counts. Genes highlighted in black represent core components of the MAPK pathway known to be regulated at the protein level through phosphorylation-based signaling (*e.g.*, Ste4, Ste11, Ste18). In contrast, genes highlighted in red represent those that appear to be regulated at the transcriptional level in response to α-factor. (**C**) Two modes of MAPK pathway regulation in response to α-factor. Schematic representation of two distinct modes of MAPK pathway regulation in *S. cerevisiae* during pheromone response. The canonical model of pheromone signaling, in which pathway components—including Ste4/Ste18, Ste20, and Ste11, Ste7—are primarily regulated at the protein level via phosphorylation (left panel). A proposed model based on the present transcriptome analysis, showing that several key components of the MAPK pathway, including STE2, FUS3, FAR1, and STE12, are additionally regulated at the gene expression level in response to α-factor (right panel). (**D**) Morphological response to α-factor in WT. Microscopic images showing the cellular morphology of WT and *sir2Δ* yeast strains following α-factor treatment. Cells were treated with α-factor at an OD_600_ of 0.2 (final concentration: 9.6 μg/ml) and incubated for 1.5 hours, followed by a second induction under the same conditions. WT cells formed characteristic shmoo projections, reflecting a normal morphological response to mating pheromone. In contrast, *sir2Δ* mutant cells failed to form shmoos, indicating impaired pheromone-induced polarization. Scale bar: 10 μm. (**E**) Heatmap of shmoo formation-related genes upregulated by α-factor in WT. Heatmap displaying the expression profiles of selected genes significantly upregulated in WT yeast following α-factor treatment, specifically those involved in shmoo formation and cell wall organization. Gene expression values are normalized and shown as Z-scores. Genes highlighted in black represent previously characterized genes known to be induced by pheromone signaling during mating-induced morphological changes. Genes highlighted in red represent novel genes from this study that were not previously associated with pheromone response, but showed significant upregulation and may contribute to shmoo formation or cell wall remodeling. (**F**) Schematic model of the localization of α-factor-induced genes associated with morphological changes. Schematic diagram illustrates the cellular localization of genes upregulated in *S. cerevisiae* upon α-factor treatment. Genes in White indicate known factors involved in cell wall remodeling and morphological changes during mating, while genes in Blue represent newly identified candidates from this study. Proteins are positioned according to their subcellular compartments, highlighting their potential roles in shmoo formation, vesicle transport, and cell fusion between mating types.

Gene Ontology (GO) analysis revealed that these upregulated genes were enriched in functional clusters related to the pheromone response. Many of these genes were associated with cell wall remodeling, consistent with the morphological changes required for mating projection (shmoo) formation (Fig. 2A).

Although previous studies have primarily focused on protein-level regulation of the MAPK signaling pathway during α-factor response, our results revealed substantial transcriptional regulation of numerous genes in response to pheromone signaling. Specifically, while key components of the MAPK cascade, including Ste4, Ste18, Ste20, Ste5, Ste11, and Ste7—are regulated post-transcriptionally, many other pheromone-induced genes showed robust transcriptional induction (>2-fold) upon α-factor treatment (Fig. 2B). These findings suggest that α-factor signaling operates through two complementary mechanisms: phosphorylation-dependent signaling and transcriptional regulation (Fig. 2C).

Furthermore, genes involved in cell wall organization and morphogenesis were significantly upregulated. Consistently, with these transcriptional changes, α-factor-treated MAT a cells displayed clear shmoo morphology (Fig. 2D). Among the upregulated genes, several known regulators of shmoo formation (marked in black, Fig. 2E) showed elevated expression. Additionally, genes involved in cell wall biogenesis or morphogenesis but not previously recognized as α-factor-responsive (marked in red, Fig. 2E) were also significantly induced. Collectively, these data indicate that α-factor-induced morphological changes, including cell wall remodeling and shmoo formation, are mediated by extensive transcriptional activation of specific target genes (Fig. 2F).

### α-Factor-Induced G1 Arrest Suppresses the Transcription of Cell Cycle, Histone, and Chromatin Organization Genes in Yeast

To investigate the transcriptional programs suppressed during the mating response, we focused on 509 genes that were significantly downregulated after α-factor stimulation. GO analysis of these 509 downregulated genes revealed a significant functional clustering of genes related to the cell cycle. In yeast, recognition of mating pheromones is known to trigger cell cycle arrest at the G1 phase, thereby preventing progression into S-phase. Consistent with this, we observed a significant downregulation of genes involved in the G1/S transition.

**Fig. 3:**
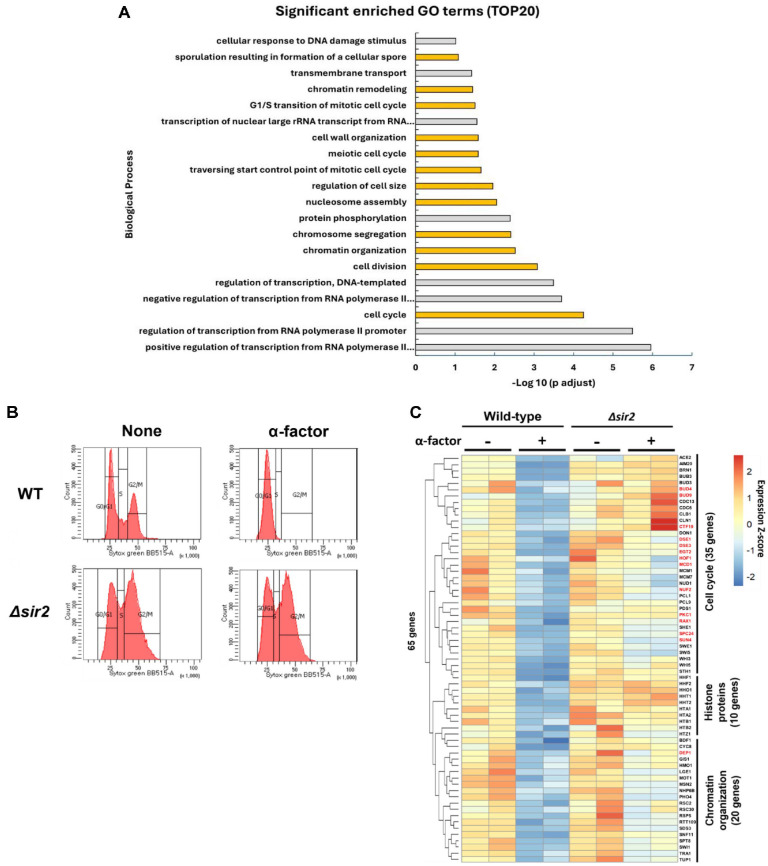
Differential gene expression and Gene Ontology of downregulated genes in WT after α-factor addition. (**A**) Gene ontology (GO) enrichment analysis of downregulated genes in WT after α-factor treatment. GO enrichment analysis was conducted using DAVID on 509 genes that were significantly downregulated in WT cells following α-factor treatment (adjusted *P* < 0.05; log_2_FC < -1). The bar plot shows the top 20 significantly enriched GO categories (biological process) based on statistical significance. The y-axis represents GO terms, and the x-axis indicates –log_10_(**P**) values. Enriched categories include cell cycle, nucleosome assembly, and chromatin organization. (**B**) cell cycle analysis by DNA contents estimation with flow cytometry in WT cells before and after α-factor treatment. Flow cytometry was performed to assess DNA content and cell cycle distribution in *S. cerevisiae* cells. Cells were collected before and after α-factor treatment (final concentration: 9.6 μg/ml; 1.5 h × 2 inductions at OD_600_ = 0.2), fixed, and stained with SYTOX Green. Histograms represent DNA content profiles, where the G1 and G2/M peaks correspond to unreplicated and replicated genomes, respectively. Top: WT cells exhibited a marked accumulation at the G1 phase following α-factor treatment, consistent with pheromone-induced G1 arrest. Bottom: In contrast, *sir2Δ* cells did not display G1 accumulation. (**C**) Heatmap of cell cycle and chromatin organization-related genes upregulated by α-factor in WT. Heatmap displaying the expression profiles of selected genes significantly upregulated in WT following α-factor treatment, specifically those involved in cell cycle, histone proteins, and chromatin organization. Gene expression values are normalized and shown as Z-scores. Representative down-regulated genes newly identified in this study are highlighted in red, consistent with the highlighting scheme used in Fig. 2.

Inhibition of the G1/S transition leads to the prevention of DNA synthesis, which consequently suppresses the synthesis of new histones. As a result, genes associated with chromatin organization and nucleosome assembly also exhibited markedly decreased expression levels (Fig. 3A). These observations indicate that α-factor-induced transcriptional changes extend beyond cell cycle control to include chromatin-related processes.

To experimentally verify whether α-factor indeed induces G1 arrest, we measured cellular DNA content using fluorescence-activated cell sorting (FACS)analysis. Before α-factor treatment, WT cells displayed a DNA content profile consistent with normal cell cycle progression. However, following α-factor treatment, WT cells were predominantly arrested in G1 phase, as evidenced by a single peak corresponding to G1-phase DNA content. In contrast, *sir2Δ*cells failed to exhibit a normal cell cycle profile regardless of α-factor treatment, indicating a disruption in proper cell cycle progression (Fig. 3B).

To further validate and visualize these transcriptional changes, we conducted heatmap analysis focusing on the previously identified downregulated genes associated with cell cycle regulation, histone genes, and chromatin organization-related genes. The heatmap clearly showed the expression patterns of 65 well-characterized genes, including 35 cell cycle regulators, 10 histone genes, and 20 chromatin organization-related genes. Notably, all of these genes displayed at least a two-fold reduction in expression in WT cells after α-factor treatment when compared to untreated conditions (Fig. 3C).

Collectively, these results strongly suggest that α-factor-induced G1 arrest substantially suppresses the transcriptional activity required for DNA replication and chromatin remodeling.

### Sir2 Is Responsible for α-Factor-Induced Gene Expression and Cell Cycle Arrest

To further investigate the regulation of pheromone-induced gene expression, we conducted RNA-seq analysis using *SIR2*-deleted yeast cells (*sir2Δ*) alongside WT cells as a control (Fig. 2B, 2E, Fig. 3C). Sir2 is a well-characterized NAD^+^-dependent histone deacetylase known for its role in transcriptional silencing at telomeres, rDNA loci, and silent mating-type regions [39-43]. During our analysis, we observed that *sir2Δ* cells exhibited defective cell cycle arrest upon α-factor treatment, a phenotype distinct from Sir2’s canonical role in heterochromatin silencing (Fig. 3B and 3C).

Specifically, genes that were significantly upregulated in wild-type cells in response to α-factor treatment failed to show similar induction in *sir2Δ* cells (Fig. 2B and 2E). In fact, many of these genes were downregulated compared to their expression in pheromone-treated WT cells. This observation suggests that Sir2 is required for the proper transcriptional activation of genes involved in the pheromone response.

Although the precise molecular mechanism underlying Sir2’s influence on pheromone signaling remains to be determined, our transcriptomic and phenotypic analyses point to several plausible indirect routes through which Sir2 may modulate the α-factor response. The loss of Sir2-mediated histone deacetylation may alter chromatin accessibility, disrupting the chromatin environment required for transcriptional aqctivation during pheromone stimulation. In addition, because Sir2 has been implicated in the G1-S transition and replication timing, defective cell-cycle control in *sir2Δ* cells may interfere with the proper execution of α-factor-induced G1 arrest. Furthermore, Sir2’s known involvement in metabolic and stress-response pathways raises the possibility that perturbed signaling crosstalk in *sir2Δ* cells indirectly imparis the transcriptional activation of pheromone-responsive genes.

These insights align with prior studies showing that Sir2 contributes to stress- or signaling-responsive transcriptional regulation, suggesting that its chromatin-regulatory capacity may extend beyond silencing to dynamic gene-expression programs activated by extracellular cues.

Together, these findings indicate that, beyond its established role in transcriptional silencing, Sir2 contributes to the transcriptional regulation necessary for the cellular response to mating pheromones. Our results reveal a previously uncharacterized function of Sir2 in facilitating the gene expression programs essential for the pheromone-induced morphological and physiological changes, including cell cycle arrest and cell wall remodeling.

## Discussion

The mating process in yeast is a highly coordinated physiological event triggered by pheromone signaling between cells of opposite mating types, leading to cell cycle arrest, morphological changes, and transcriptional reprogramming. In this study, we comprehensively characterized for the first time the genome-wide transcriptional responses of WT yeast cells before and after α-factor treatment using RNA-seq. Our RNA-seq analysis identified 957 genes whose expression was significantly altered upon α-factor treatment, comprising 448 upregulated genes and 509 downregulated genes.

One of the most notable findings from our study is the discovery that certain components of the MAPK signaling pathway are transcriptionally regulated in response to pheromone stimulation. While previous studies have predominantly focused on phosphorylation-dependent mechanisms as the principal regulatory mode for MAPK signaling during the mating response, our data suggest that transcriptional regulation at the RNA level also plays a significant role. Specifically, the expression levels of some MAPK pathway genes are dynamically modulated following α-factor exposure, highlighting a previously underappreciated layer of regulation within this signaling cascade. This transcriptional modulation may serve to fine-tune the amplitude or duration of the signaling response. Given this novel insight, further studies are warranted to elucidate which transcription factors or chromatin remodeling factors mediate this RNA-level regulation during pheromone signaling, and how these factors integrate with canonical phosphorylation-based mechanisms to ensure proper cellular outcomes.

Additionally, our RNA-seq data revealed that genes involved in cell cycle progression, histone biosynthesis, chromatin remodeling, and nucleosome assembly are significantly downregulated following α-factor treatment. These findings align well with the established biological response of G1 phase arrest during mating, wherein cells halt DNA replication and suppress histone gene expression, which naturally leads to reduced nucleosome assembly and chromatin remodeling activities. This global transcriptional suppression of chromatin-related genes further reinforces the notion that cells actively remodel their transcriptional landscape to accommodate G1 arrest. Our FACS analysis of DNA content corroborated these transcriptomic data, showing that WT cells arrest at G1 upon α-factor treatment, while this arrest was notably absent in *sir2Δ* mutant cells. These observations collectively suggest a strong linkage between pheromone-induced signaling, transcriptional regulation, and cell cycle control mechanisms. Future studies should address the precise molecular mechanisms that regulate chromatin remodeling and nucleosome assembly upon pheromone signaling, as well as investigate how the transcriptional silencing of these genes is reversed during re-entry into the mitotic cell cycle.

Importantly, we also uncovered an unexpected and novel role for Sir2 in transcriptional regulation during the pheromone response. Sir2 has long been recognized for its role as a NAD^+^-dependent histone deacetylase involved in transcriptional silencing at specific genomic regions, such as telomeres, rDNA, and mating-type loci [39-43]. However, our analysis using the *sir2Δ* mutant revealed that cells lacking Sir2 exhibit defective cell cycle responses to α-factor, failing to arrest properly at G1. Moreover, genes that were normally upregulated in WT cells following α-factor treatment were paradoxically downregulated *sir2Δ* cells. This striking phenotype suggests that Sir2 contributes to the regulation of pheromone-induced gene expression and cell cycle arrest through mechanisms beyond its classical silencing role.

Previous studies have suggested that Sir2 can influence chromatin accessibility and transcriptional competence beyond heterochromatic regions, particularly under metabolic or stress conditions [39, 44-49]. Sir2-dependent deacetylation of histones contributes to proper nucleosome positioning and promoter architecture, which are essential for the dynamic recruitment of transcription factors. It is therefore plausible that, in the absence of Sir2, excessive histone acetylation and altered nucleosome occupancy hinder the binding of key transcriptional activators such as Ste12 and Tec1, leading to the reduced induction of pheromone-responsive genes. In addition, Sir2 has been implicated in cell-cycle regulation through its impact on G1–S cyclin expression and replication timing [47, 50], providing another layer of connection between Sir2 and pheromone-induced G1 arrest. Moreover, Sir2’s roles in stress- and metabolism-related signaling pathways-including NAD^+^ homeostasis, oxidative stress responses, and nutrient-sensing mechanisms-may indirectly influence transcriptional programs responding to external cues such as mating pheromones. Together, these considerations support a model in which Sir2 functions as a chromatin-based integrator that coordinates cell-cycle progression, metabolic state, and signaling-dependent transcriptional reprogramming during the pheromone response.

While this study provides a comprehensive transcriptomic profile of WT yeast cells responding to pheromone signaling, it also highlights the need for broader comparative analyses. For example, future studies could extend these findings by examining additional mutant strains involved in the mating pathway or comparing responses between different mating-type cells. Such analyses would offer deeper insights into the conserved and divergent aspects of the transcriptional networks orchestrating pheromone responses and contribute to our understanding of how these networks are tailored to specific physiological contexts.

In summary, our study provides the first comprehensive transcriptomic analysis of yeast pheromone responses and reveals that transcriptional regulation of MAPK pathway components operates alongside classical phosphorylation-based mechanisms. Furthermore, our findings propose a novel function for Sir2 in regulating transcriptional responses to pheromone signaling and cell cycle arrest, expanding the known roles of this key chromatin regulator. These insights enhance our understanding of the complex regulatory networks governing mating responses and cell cycle control in yeast and may provide broader implications for the study of pheromone signaling and cell cycle regulation in other eukaryotic systems.

## Supplemental Materials

Supplementary data for this paper are available on-line only at http://jmb.or.kr.
